# Irisin, in women and men: blood pressure, heart rate, obesity and insulin resistance

**DOI:** 10.3389/fendo.2023.1193110

**Published:** 2023-06-28

**Authors:** Delia Almeida González, María del Cristo Rodríguez-Pérez, Manuel Fuentes Ferrer, Francisco Javier Cuevas Fernández, Itahisa Marcelino Rodríguez, Antonio Cabrera de León

**Affiliations:** ^1^ Immunology Section, Nuestra Señora de Candelaria University Hospital, Santa Cruz de Tenerife, Canary Island, Spain; ^2^ Research Unit, Nuestra Señora de Candelaria University Hospital and Primary Care, Santa Cruz de Tenerife, Canary Islands, Spain; ^3^ Barranco Grande Health Center, Primary Care, Santa Cruz de Tenerife, Canary Island, Spain; ^4^ Public Health and Preventive Medicine Department, La Laguna University, Santa Cruz de Tenerife, Canary Islands, Spain

**Keywords:** irisin, blood pressure, heart rate, insulin resistance, obesity

## Abstract

**Background:**

Irisin is a myokine that increases with leisure time physical activity (LTPA) and for which a cardiovascular protective role has been postulated. Our aim was to assess this role in the general population.

**Methods:**

A cross-sectional analysis was performed in a large randomly selected population sample (n=2298 women and 1529 men). Apart from age and sex, we record anthropometrics (blood pressure, heart rate, obesity), lifestyle (LTPA, smoking, alcohol), and biochemical measurements (irisin, lipid profile, insulin resistance). Correlations and regression multivariate models were used to analyze the association of irisin levels with the studied factors.

**Results:**

The variables more strongly and directly associated with irisin, adjusting the studied factors separately in women and men, were HOMA-2 (p=0.043 and p=0.001, respectively) and LTPA (p<0.001 and p=0.001, respectively). Also heart rate inversely (p=0.005 and p=0.002, respectively) and DBP directly (p<0.005 and p=0.045, respectively) were associated to irisin in both sexes. The waist/height ratio (p<0.001) was inversely associated to irisin only in women, and the alcohol drinking was directly associated (p=0.029) only in men.

**Conclusion:**

We provide new findings for irisin, such as its association with DBP and with heart rate; furthermore, in women irisin is associated to abdominal obesity, and in men is associated to the alcohol intake. We also corroborate the association of irisin with LTPA and insulin resistance. The associations detected point towards a protective role of irisin in the maintenance of cardiometabolic health.

## 1 Introduction

Despite the noteworthy advances in its prevention and treatment, the cardiovascular diseases are still the leading cause of mortality in the world, and their decline has slowed in the most developed countries (1). Health efforts in early diagnosis have led to the study of new biomarkers associated with these diseases, such as cytokines. In particular, myokines participate in the communication between muscle, adipose tissue and other organs, intervening in the control of body weight and energy metabolism (2–4).

Irisin is a myokine which was discovered less than 10 years ago, and it constitutes the cleaved and secreted fragment of fibronectin type III, specifically the *FNDC5* domain (5). Most of it is synthesized by skeletal muscle myocytes, and there is a direct association between leisure time physical activity (LTPA) and serum irisin levels in the general adult population (6). Furthermore, it has been reported that it participates in glucose metabolism promoting insulin sensitivity, glucose absorption, glycogenolysis and reduction of gluconeogenesis (7, 8). Some anti-inflammatory and anti-apoptotic effect of irisin have been postulated (9), and, because of this, irisin has been suggested as a therapeutic target in cardiovascular diseases (10).

Although much remains to be clarified regarding its physiology, there are enough studies to accept that irisin is involved in the maintenance of cardiovascular health, and this myokine has a protective role and even a potential therapeutic use on low-grade inflammation. However, some findings on the effects of serum irisin levels have been inconsistent, and the very existence of the molecule has been questioned (11); certain methodological problems related to its plasma measurement and the technical variability of the main commercial kits have been pointed out, but the existence of this myokine has been demonstrated (12). Yet, in spite of more than a thousand publications regarding the physiological effects of irisin to date, there is a degree of disparity in the results that may be due in part to the small size of the analyzed samples in most of the studies.

The aim of the study was to analyze, in a large sample of general population, and under real-life conditions, the relationship between serum irisin concentration and some well-known cardiovascular risk factors.

## 2 Materials and methods

### 2.1 Study design and setting

A cross-sectional analysis of the relationship between irisin and the prevalence of cardiovascular risk factors was conducted in 3,827 participants enrolled in the general adult population cohort called “CDC of the Canary Islands”. All participants gave their informed consent and the project was evaluated and approved by the Ethics Committee of the University Hospital Nuestra Señora de Candelaria.

### 2.2 Subjects

The “CDC de Canarias” cohort (n=6729) was recruited from the general population of the Canary Islands (Spain). Its methodology was described in detail (13). Briefly, the criteria for inclusion were: 1) To be in the census, 2) To be 18-75 years old, and 3) To give her or his informed consent. The only exclusion criteria was the inability to respond to the interviewer on their own and not having a proxy to do it. Each participant underwent a physical examination and, subsequently, trained interviewers administered a questionnaire about their lifestyle (tobacco, alcohol, physical activity). Furthermore, a fasting venous blood sample was extracted early in the morning to determine biochemical parameters (serum lipids, blood glucose levels, etc.) and serum aliquots were stored at -80°C.

### 2.3 Measure of irisin

In order to measure irisin (µg/mL), a serum aliquot stored at -80°C from recruitment was thawed and the irisin concentration was determined with ELISA kits (RAG018R. BioVendor, Brno, Czech Republic). The samples were run diluted so that the irisin concentration was within the assay range (0.001 µg/ml - 5 µg/ml). The intra-assay and inter-assay coefficients of variation were 20.2% and 35.1%, respectively. In order to obtain these values, a serum pool of 600 patients from the immunology laboratory was prepared, which were aliquoted and kept at -80°C until use. The determinations were made with a Triturus autoanalyzer (Grifols, Barcelona, Spain), in the Immunology laboratory. The same aliquot and procedures were used to measure C-peptide concentration by enzyme immunoanalysis (Biosource^®^, ng/mL, intra-assay coefficient of variation 3.98%, inter-assay coefficient of variation 11.86%). For funding reasons, these measurements were not made in all the cohort, but in a representative randomly selected sample of it (n = 3,827).

### 2.4 Cardiovascular risk factors

The anthropometric measurements recorded in the recruitment were the body mass index (BMI, Kg/m^2^), waist-to- height ratio, systolic (SBP) and diastolic (DBP) blood pressure (mmHg), and heart rate in beats/minute (b/m). In regard to biochemical factors (mg/dL), serum glucose, HDL-cholesterol, LDL-cholesterol and triglycerides were measured. Apart from this, the status of insulin resistance was measured with the ratio triglycerides/HDL-cholesterol, and also with the HOMA-2 model which combines serum concentrations of glucose and C-peptide.

### 2.5 Lifestyle data

Information on physical activity was recorded during the enrollment interview with each participant, and the total time spent in LTPA throughout the week was estimated in hours/week (h/w); this variable involved all types of PA including recreational, sports, domestic or active transport activities. Regarding the tobacco consumption, it was registered the declared number of years actively smoking. Finally, alcohol consumption in grams/day was taken from the diet questionnaire of the “CDC of the Canary Islands” study.

### 2.6 Statistical analysis

Continuous variables were summarized with their mean ± standard deviation. The bivariate association of irisin with every variable was analyzed, separately for women and men, using the Pearson correlation coefficient. With the exception of irisin, which was measured for this study in the 3827 aforementioned subjects, the information on the other study variables was available for all the subjects in the entire cohort.

For the multivariate analysis, 14 linear regression multivariate models were adjusted for age and LTPA in each sex, with irisin as the dependent variable. Finally, a linear regression model (backward method) in each sex was adjusted for age, waist/height ratio, heart rate, LDL-cholesterol, triglycerides/HDL-cholesterol ratio, HOMA-2, SBP, DBP, LTPA, smoking and alcohol. To avoid collinearity, the values of C-peptide, glucose, HDL-cholesterol, and triglycerides were not included in this model; also, only one index of obesity (Waist/height ratio) was included. All the models were summarized with their regression coefficients (RC) and 95% confidence intervals (95% CI).

Statistical analyses were performed using SPSS statistical software (version 26.0 for Windows; SPSS Inc., Chicago, IL). We used two-tailed hypothesis contrast tests.

## 3 Results

The studied sample consisted of 3,827 adults recruited from the general population. The mean irisin concentration in the participants was 33.9 ± 14.4 μg/ml, and its normal range was between 5.1 and 62.7 μg/ml. **Table 1** shows that irisin values were higher in women (p<0.001) and it summarizes the distribution of the other variables in each sex.

**Table 1:** Baseline characteristics of the study participants in women and men.

	Women n=2298	Men n=1529	p
Irisin. µg/mL	34.97 ± 14.52	32.31 ± 14.02	<0.001
Age. years	42.92 ± 12.89	42.80 ± 12.84	0.699
BMI. Kg/m^2^	27.33 ± 5.54	27.52 ± 4.31	0.110
Waist/height ratio	54.25 ± 13.15	55.48 ± 7.62	<0.001
Heart rate. b/m	74.85 ± 10.23	71.19 ± 10.96	<0.001
LDL cholesterol. mg/dL	125.65 ± 35.78	130.29 ± 36.92	<0.001
HDL-cholesterol. mg/dL	54.69 ± 13.14	46.43 ± 12.36	<0.001
Triglycerides. mg/dL	108.21 ± 79.92	140.90 ± 101.42	<0.001
Triglycerides/HDL ratio	2.24 ± 2.48	3.47 ± 3.34	<0.001
Blood glucose. mg/dL	93.89 ± 23.89	100.23 ± 27.32	<0.001
C-peptide. ng/mL	2.12 ± 1.17	2.21 ± 1.31	<0.001
HOMA-2	0.50 ± 0.36	0.56 ± 0.41	<0.001
SBP. mmHg	119.95 ± 19.40	127.11 ± 17.63	<0.001
DBP. mmHg	75.48 ± 11.21	80.74 ± 10.83	<0.001
Physical activity. h/wk	1.43 ± 1.60	1.29 ± 1.67	0.001
Tobacco. Years smoking	16.64 ± 9.72	21.83 ± 12.48	<0.001
Alcohol consumption. gr/day	2.33 ± 6.10	18.31 ± 31.14	<0.001

SBP, Systolic blood pressure; DBP, Diastolic blood pressure.

**Table 2** shows, in women and men, the correlation coefficients between the irisin values and the studied variables. In women, the strongest associations with irisin were those of HDL-cholesterol (r=0.164; p<0.001), waist/height ratio (r=-0.137; p<0.001), BMI (r=-0.123; p<0.001), LTPA (r=0.120; p<0.001), and age (r=-0.117; p<0.001); other associated variables were years smoking (r=-0.107; p=0.004) and SBP (r=-0.075; p<0.001). In men, the strongest association were for LTPA (r=0,098; p<0.001) and HOMA-2 (r=0.089; p=0.001), with no association for HDL-cholesterol, obesity indexes and age; other associated variables in men were C-peptide (r=0.074; p=0.005) DBP (r=0.069; p=0.007) and alcohol intake (r=0.058; p=0.028). Apart from LTPA, only the heart rate correlated with irisin in both sexes (r=-0.058; p=0.006 in women, and r=-0.067; p=0.009 in men).

**Table 2:** Correlation coefficients of serum irisin levels with cardiovascular risk factors in women and men.

	Irisin (µg/mL) in Women n=2298	Irisin (µg/mL) in Men n=1529
Age. years	**-0.117; p<0.001**	-0.003; p=0.912
BMI. Kg/m^2^	**-0.123; p<0.001**	0.016; p=0.526
Waist/height ratio	**-0.137; p<0.001**	0.039; p=0.144
Heart rate. b/m	**-0.058; p=0.006**	**-0.067; p=0.009**
LDL cholesterol. mg/dL	**-0.074; p=0.001**	-0.007; p=0.793
HDL-cholesterol. mg/dL	**0.164; p<0.001**	0.034; p=0.196
Triglycerides. mg/dL	-0.001; p=0.979	0.010; p=0.717
Triglycerides/HDL ratio	-0.03; p= 0.175	-0.00; p=0.959
Blood glucose. mg/dL	-0.010; p=0.654	0.042; p=0.114
C-peptide. ng/mL	-0.026; p=0.222	**0.074; p=0.005**
HOMA-2	-0.020; p=0.355	**0.089; p=0.001**
SBP. mmHg	**-0.075; p<0.001**	0.043; p=0.093
DBP. mmHg	-0.029; p=0.166	**0.069; p=0.007**
Physical activity. h/week	**0.120; p<0.001**	**0.098; p<0.001**
Tobacco. Years smoking	**-0.107; p=0.004**	-0.010; p=0.777
Alcohol consumption. gr/day	0.016; p=0.465	**0.058; p=0.028**

SBP, Systolic blood pressure; DBP, Diastolic blood pressure; significant values in bold.

The multivariate analysis summarized in **Table 3** shows that, adjusting for age and LTPA, in women the only variables associated to irisin were the BMI (RC= -0.21; p<0.001), waist/height ratio (RC=-0.15; p<0.001), HDL-cholesterol (RC=0.17; p<0.001) and heart rate (RC=-0.07; p=0.022). However, in men the associated variables were HOMA-2 (RC=3.43; p<0.001), C-peptide (RC=0.86; p=0.004), DBP (RC=0.09; p=0.012), heart rate (RC=-0.08; p=0.029) and alcohol intake (RC=0.03; p=0.037).The final multivariate analysis (**Table 4**), presents the variables associated to irisin in each sex when all the studied variables were adjusted in a single model. The variables more strongly associated with irisin, in women and men, were HOMA-2 (RC=2.07 [p=0.043] and 3.40 [p=0.001], respectively) and LTPA (RC=1.58 [p<0.001] and 1.01[p=0.001], respectively); heart rate and DBP were also associated to irisin in both sexes. The waist/height ratio was retained in the model only in women, and the alcohol drinking was retained only in men; the age was retained in both sexes, although it reached significance only in women.

**Table 3:** Each line resumes a linear multivariable regression model for women and another one for men, with the regression coefficients (CI95%) for the independent variable adjusted by age and physical activity.

INDEPENDENT VARIABLE	DEPENDENT VARIABLE Irisin WOMEN n=2298 RC (CI95%); p	DEPENDENT VARIABLE Irisin MEN n=1529 RC (CI95%); p
BMI. Kg/m^2^	**-0.21 (-0.33- -0.10); p<0.001**	0.07 (-0.11- 0.25); p=0.442
Waist/height ratio	-**0.15 (-0.21- -0.09); p<0.001**	0.09 (-0.02- 0.20); p=0.105
Heart rate. b/m	**-0.07 (-0.13- -0.01); p=0.022**	**-0.08 (-0.14- -0.01); p=0.029**
LDL-cholesterol. mg/dL	-0.01 (-0.03- 0.00); p=0.138	-0.00 (-0.02- 0.02); p=0.821
HDL-cholesterol. mg/dL	**0.17 (0.12- 0.21); p<0.001**	0.04 (-0.02- 0.10); p=0.226
Triglycerides. mg/dL	0.01 (-0.00- 0.02); p=0.138	0.00 (-0.01- 0.01); p=0.620
Triglycerides/HDL ratio	-0.03 (-0.35- 0.28); p=0.845	0.01 (-0.23- 0.24); p=0.061
Blood glucose. mg/dL	0.02 (-0.01- 0.05); p=0.187	0.02 (-0.01- 0.05); p=0.108
C-peptide. ng/mL	-0.06 (-0.58- 0.47); p=0.831	**0.86 (0.28- 1.44); p=0.004**
HOMA-2	0.64 (-1.23- 2.53); p=0.510	**3.43 (1.52- 5.34); p<0.001**
SBP. mmHg	-0.00 (-0.04- 0.03); p=0.835	0.05 (-0.00- 0.09); p=0.069
DBP. mmHg	0.04 (-0.19- 0.10); p=0.179	**0.09 (0.02- 0.16); p=0.012**
Tobacco. Years smoking	0.03 (-0.04- 0.09); p=0.430	-0.00 (-0.06- 0.06); p=0.958
Alcohol. gr/day	0.19 (-0.72- 0.11); p=0.678	**0.03 (0.00- 0.05); p=0.037**

SBP, Systolic blood pressure; DBP, Diastolic blood pressure; significant values in bold. The dependent variable was irisin (µg/mL) in all the models.

**Table 4:** Final linear regression model: Variables retained when adjusting in a single model (backward method), for each sex, the variables: age, waist/height ratio, heart rate, LDL, cholesterol, triglycerides/HDL ratio, HOMA-2, SBP, DBP, physical activity, smoking and alcohol.

INDEPENDENT VARIABLES	DEPENDENT VARIABLE Irisin (µg/mL) WOMEN n=2298 RC (CI95%); p	INDEPENDENT VARIABLES	DEPENDENT VARIABLE Irisin (µg/mL) MEN n=1529 RC (CI95%); p
Age. years	-0.12 (-0.18- -0.06); p<0.001	Age. years	-0.05 (-0.12- 0.01); p=0.097
Waist/height ratio	-0.19 (-0.26- -0.13); p<0.001	Heart rate. b/m	-0.11 (-0.18- -0.04); p=0.002
Heart rate. b/m	-0.09 (-0.15- -0.03); p=0.005	HOMA-2	3.40 (1.45- 5.36); p=0.001
HOMA-2	2.07 (0.06- 4.07); p=0.043	DBP. mmHg	0.08 (0.00- 0.15); p=0.045
DBP. mmHg	0.09 (0.03- 0.15); p=0.005	Physical activity. h/w	1.01 (0.41- 1.62); p=0.001
Physical activity. h/w	1.58 (1.06- 2.10); p<0.001	Alcohol. gr/day	0.03 (0.00- 0.06); p=0.029

DBP, Diastolic blood pressure.

## 4 Discussion

In a large sample of general population, the present study has found a direct association of irisin with DBP and an inverse association with heart rate; also, we show an inverse association between irisin and abdominal obesity only in women, and a direct association between irisin and alcohol intake only in men. The study corroborates the association of irisin with LTPA and HOMA-2.

Being a myokine that is released during skeletal muscle contraction, irisin is considered an exerkine, mainly produced during adaptation to exercise (14). The effect on this molecule was initially described when physical exercise was done intensely and acutely (15). In fact, elite athletes have higher baseline levels of irisin than the general population (16). However, its level depends on environmental factors too (17), and the positive association of LTPA with irisin is well stablished and it is stronger than the association between LTPA and some known biomarkers of physical activity like HDL-cholesterol or resistin (6). In the present study, we have confirmed the robust and independent association between the concentration of irisin and the levels of LTPA, which includes recreational, sports, domestic or active transport activities; we think that focusing on LTPA provides a good estimation of irisin levels in the real-life conditions of most people. In fact, the beneficial effects of LTPA on cardiovascular heath are attributed to the reduction in heart rate and blood pressure (18), and both parameters are related to irisin in our study.

The estimation of insulin resistance with the HOMA-2 model has been repeatedly used to demonstrate its association with irisin (7, 8). We found that the higher the level of serum irisin, the higher the level of HOMA-2. Although the effect of irisin on the endocrine pancreas *in vitro* and ex vivo has not been fully investigated, most publications, including meta-analysis, concluded that circulating irisin is directly and positively associated with insulin resistance in non-diabetic adults, suggesting that the irisin is involved in the regulation of pancreas and in the enhancement of insulin sensitivity (19). In animal models, it has also been proven that the overexpression of *FNDC5* increases the serum levels of irisin and improves hyperglycemia and hyperinsulinemia, leading in an improvement in the insulin resistance of the mice (5). Hence, we are corroborating here the irisin-insulin resistance association in both sexes independently of key factors like age, physical activity and obesity, which could highlight the crosstalk between muscle and glucose metabolism. We speculate that the increase of the irisin concentrations in an insulin resistance microenvironment might be a compensatory mechanism to attempt the recovery of the glucose homeostasis, promoting the glucose uptake and consequently, preventing the development of type 2 diabetes. When diabetes occurs is usual to find low irisin concentrations, suggesting that glucose may be a critical suppressor of irisin synthesis in skeletal muscle, but the insulin resistance is a previous step in impaired glucose metabolism (20). However, the detected association in our study require new approaches of the impairment of glucose metabolism through of longitudinal follow-up studies.

To the best of our knowledge, we have measured irisin here in the largest general population sample studied to date and we have found a direct association between serum irisin levels and DBP. Although the bivariate analysis showed an association of SBP with irisin in men, it was not corroborated as soon as it was adjusted by LTPA and age. So, in women and men, the only blood pressure measurement related with irisin was DBP. Even though previous studies have described systolic and diastolic blood pressure correlations with irisin, they did not adjust physical activity and this is crucial for this exerkine; in addition their sample sizes were small and the authors recognize the need of future studies with larger sample sizes (21, 22). It has been suggested that irisin plays a role in blood pressure control, perhaps acting as cofactor to modulate vascular function, so a balanced serum level of irisin would be essential in maintaining vascular tone (23). In any case, we have shown that in the general population only DBP is associated with irisin in both sexes regardless of age, physical activity, and insulin resistance.

As far as we know, the inverse association that we have found between irisin and heart rate has not been described before in large observational studies. Even more, the only study that has assessed this association included just seventeen adults to perform an incremental exhaustive exercise and they did not observe significant association of irisin with heart rate at rest, peak, or recovery (24). Obviously, the small sample size implies lack of power and impedes a stratified analysis into subgroups. Therefore, when the studied sample is big enough it can be verified the existence of an inverse relationship of irisin and heart rate in women and men. This association was detected since the initial bivariate analysis and it was corroborated in the multivariate models, and it suggests that irisin can be involved in the autonomic nervous system regulation, which could give this myokine a protective role for cardiovascular health.

Interestingly, obesity was associated with irisin only in women. This inverse association was present since the bivariate to the multivariate analysis, even when we used different obesity indexes, such as waist/height ratio for abdominal obesity, or BMI for general obesity. The sex differences in the body fat distribution have been studied for a long time (25), and continue to be an object of interest today (26). These differences do not seem to act on insulin resistance (25), and in fact we detected the irisin relationship with HOMA-2 in both sexes. Unfortunately we did not have a measure to differentiate the lean body weight of the skeletal muscle mass, and we don’t know if serum irisin is more related to any of them, but in previous studies myokines have not shown association with bone parameters (27). Some studies suggest that sex differences in the adiposity distribution play a role in the different cardiometabolic risk of men and women (28), but an exact role for irisin in this issue would need future studies. Being a product of the skeletal muscle, is not strange that serum irisin levels are different between sexes; however, in spite that women have much less skeletal muscle mass, their irisin values are higher than those of men. This is still a novel myokine, the production and effects of which are not well known.

Finally, we have not found any study describing the direct association of irisin with alcohol intake that we have detected in men. As an average, the intake of alcohol in men multiplied by 9 the consumption declared by women. So, may be the described association is only present when alcohol levels are above some limits or, as it happens with HDL-cholesterol, there is different relationship of alcohol with biomolecules in women and men (29).

Our study has limitations mainly based on its cross-sectional design, which does not allow to detect causal associations. Furthermore, the authors also recognize the limitations inherent to studies in which some information is obtained through questionnaires, although the validity of this method has been widely demonstrated. To minimize these limitations we used validated questionnaires and assessment tools for recording of alcohol intake, smoking, physical activity, and anthropometric measurements; to reduce bias from laboratory results, previously validated and specific assays were run blindly and under code by specialized laboratory personnel; moreover, any random measurement error should lead to suppress statistical significance toward the null and, thus, could not have altered the statistically significant results presented here. Also, since we studied a sample of the general population in their usual living conditions, it was not impossible that some person would have exercised before the blood draw, and so increasing his or her irisin level; however, the fasting venous blood sample was drawn early in the morning and under fasting conditions, and our results are consistent with previous findings of the association of irisin with age, physical activity and insulin resistance, so the authors think that there is not bias. This study has also some strengths, as the large sample size, in addition to not studying patients but participants randomly selected from the general population under normal living conditions.

In summary, this study analyzed the relationship of some cardiometabolic risk factors with the concentration of irisin in a large size of the general population. We conclude providing new findings for irisin, such as its direct association with DBP and its inverse association with heart rate; furthermore, in women irisin is inversely associated to abdominal obesity, and in men is directly associated to the alcohol intake. We also corroborate the association of irisin with LTPA and insulin resistance. The associations detected point towards a protective role of irisin in the maintenance of cardiometabolic health.

## Data availability statement

The raw data supporting the conclusions of this article will be made available by the authors, without undue reservation.

## Ethics statement

The studies involving human participants were reviewed and approved by Ethics Committee of the University Hospital Nuestra Señora de Candelaria. The patients/participants provided their written informed consent to participate in this study.

## Author contributions

All the authors have taken part in the study and in the writing of the article, and we can take public responsibility for it. AC and DA planned the original study and wrote the final manuscript. DA directed all the laboratory procedures and quality control of the data. MCR and MF drafted the manuscript. IM and FJC analyzed the data and revised the manuscript. All authors contributed to study design, and accepted the final version of the manuscript.
